# Antimicrobial Stewardship Program in an acute care hospital in New Orleans: impact on antibiotic utilization

**DOI:** 10.1017/ash.2025.10284

**Published:** 2026-04-01

**Authors:** Michelle D. Blyth, Bionca W. Conway, Lonzie Beamon, M. Jacques Nsuami, Julio E. Figueroa

**Affiliations:** 1 Section of Infectious Diseases, Department of Internal Medicine, School of Medicine, Louisiana State University Health Sciences Center, New Orleans, LA, USA; 2 LCMC Health, Touro Infirmary, New Orleans, LA, USA

## Abstract

**Objectives:**

To evaluate antibiotic utilization in relation to antimicrobial stewardship activities in a 170-bed Touro Infirmary hospital in New Orleans.

**Methods:**

We evaluated antibiotic utilization for antipseudomonal beta-lactams, methicillin-resistant *Staphylococcus aureus* antibiotics (anti-MRSA), fluoroquinolones, and ceftriaxone in relation to antimicrobial stewardship activities that were implemented at the hospital and monitored between August 1, 2018, and August 31, 2025. We recorded days of antibiotic therapy and days of intravenous antibiotic administration for 19-months baseline, during 10 months of COVID-19-dictated antibiotic utilization, and during 14 months of MRSA PCR testing at Touro (Antimicrobial Stewardship Intervention [ASI] 1), 20 months of blood culture identification (BCID) at Touro (ASI 2, time 1), followed by 22 months of BCID and other cultures sent out to University Medical Center (ASI 2, time 2).

**Results:**

During the observation period, days of therapy for antipseudomonal beta-lactams, anti-MRSA, and fluoroquinolones consistently and significantly decreased from baseline to ASI 2, time 2 (*P* < .0001 for each decrease). Days of therapy for ceftriaxone significantly increased from ASI 1 to ASI 2, time 2 (*P* < .0001). Days of therapy for all antibiotics consistently decreased from baseline to ASI 2, time 1 (*P* < .0001) but not through time 2 (*P* = .571). Days of intravenous administration of antibiotics significantly decreased from baseline to the COVID-19 period (*P* = .002) and decreased again significantly from ASI 1 to ASI 2, time 1 (*P* = .003).

**Conclusions:**

Clinical microbiology laboratories integrated into Antimicrobial Stewardship Program are associated with reductions in days of antibiotic therapy and days of intravenous antibiotic administration at Touro Infirmary.

## Introduction

Since the discovery of penicillin in 1928, advances in antibiotics have revolutionized the management of infectious diseases, improving survival and longevity.^1–4^ However, the use of antibiotics presents a duality: while improving and saving lives, improper use contributes to adverse events, promotes antimicrobial resistance, and imposes a consequential threat to public health.^5–11^

In 2014, the Centers for Disease Control and Prevention (CDC) petitioned all federally subsidized US hospitals to implement antimicrobial stewardship programs (ASPs) through the release of seven core elements aimed to provide a framework for combating antimicrobial misuse and resistance.^12^ The seven core elements, updated in 2019, are (1) Hospital Leadership Commitment, (2) Accountability, (3) Pharmacy Expertise, (4) Action, (5) Tracking, (6) Reporting, and (7) Education.^12^

Clinical microbiology laboratories integrated into ASPs are associated with improved patient outcomes and reduced hospital costs.^13–15^ Patient outcomes that have shown improvement with the integration of clinical microbiology laboratories into ASPs include reduction in time to optimal antimicrobial therapy,^16–21^ decreased time of total antibiotic exposure and an early discontinuation of unnecessary antibiotic therapy,^22^ reduction in antibiotic use,^23^ reduction in hospital or infection-related length of stay,^18–20,22^ and decreased mortality.^20,21,23^ Some of these studies also reported reductions in hospital or infection-related costs.^18,19,21,22^

Touro Infirmary is a community-based, not-for-profit, faith-based hospital in New Orleans, Louisiana. Founded in 1852, it has evolved into primarily an acute care hospital with modern facilities that utilize the latest technology. The hospital is currently part of the Louisiana Children’s Medical Center (LCMC) Health system; it has 170 beds, one medical intensive care unit, one surgical intensive care unit, inpatient medical/surgical wards, an oncology ward, a geriatrics ward, and a neurorehabilitation unit. Although there were multiple starts to AS activities, a team of ASP led by an infectious disease medical director and a clinical pharmacist with infectious disease training was solidly established at the hospital, with the commitment of the hospital leadership, in 2017.^12,24^ The clinical pharmacist with infectious disease training is the lead pharmacist who develops most of the protocols designed to promote judicious and appropriate use of antibiotics at the hospital. This pharmacist leads a team of other clinical pharmacists who work at the different units/wards of the hospital, and part of their AS task is to look at opportunities for adjusting antibiotic levels in their patient areas.

The AS activities of the ASP at Touro (CDC’s core element #4)^12^ are regularly reported to prescribers, pharmacists, nurses, and hospital leadership for their education, but their impact has never been evaluated. In this study, we report on the utilization of selected antimicrobial agents at Touro in relation to AS activities that were implemented and monitored between August 1, 2018, and August 31, 2025. During the study period, we evaluated days of antibiotic therapy and days of intravenous antibiotic administration among all patients present at the hospital.^23^ We hypothesized that days of antibiotic therapy and of intravenous antibiotic administration would decrease with the AS activities. Because ceftriaxone was the institution’s de-escalation drug during the study period, we expected that days of its utilization would not decrease with the AS activities.

## Methods

### Sources of study data

The Touro Infirmary ASP benefits from the support of the hospital’s latest laboratory capabilities and of an integrated computerized information system.^12,24,25^ The hospital uses EPIC, with a module which integrates calculators that determine the numbers of antibiotic doses that are administered daily in different units/wards of the hospital and the total number of patient-days present in the hospital.

### Antimicrobial stewardship activities and monitoring of antibiotic utilization

Since the ASP was established at Touro, pharmacists perform weekday reviews of all patients on vancomycin, and the pharmacy operates a vancomycin pharmacokinetic service that constantly monitors and adjusts the levels of vancomycin administered to patients in the hospital. The use of antipseudomonal beta-lactams is also reviewed on weekdays for the appropriateness of each use. Fluoroquinolones are not reviewed daily but are targeted for education and feedback, with the goal of reducing their overall use in the hospital. Monthly meetings are held with providers as well as regular meetings with the medical staff of the hospital, during which AS activities are discussed for their education.

Monthly monitoring of antibiotic utilization at Touro began on August 1, 2018, and continued for 19 months until February 29, 2020, when the COVID-19 pandemic disrupted all patterns of patient hospitalizations and antibiotic utilization at the hospital (baseline period). From March 1, 2020, to December 31, 2020 (10 months), antibiotic utilization that was dictated by the COVID-19 pandemic was monitored. Beginning January 1, 2021, tracking of AS interventions at Touro started.

### Antimicrobial stewardship interventions (ASIs)

#### ASI 1

From January 1, 2021, to February 28, 2022 (14 months), a pharmacy-driven nares MRSA polymerase chain reaction (PCR) testing protocol between the hospital’s laboratory and the pharmacy was established and monitored. The laboratory had brought an MRSA PCR test designed for rapid detection of *S. aureus* target DNA and the genes for methicillin resistance (*mec*A/C) from nasal swabs obtained from patients at risk of nasal *S. aureus* colonization (Cepheid Xpert® SA Nasal Complete, Sunnyvale, CA, USA, 2019). With this laboratory capability, the ASP passed a policy that allowed the pharmacist to order this test for patients receiving vancomycin.

#### ASI 2

Blood culture identification (BCID) was a laboratory protocol under which a rapid multiplex PCR-based testing of positive blood cultures was performed for the diagnosis of microorganisms present in the bloodstream.^26^ Touro introduced the FDA-cleared BioFire® FilmArray® BCID2 panel testing (BioMérieux, BioFire Diagnostics, Salt Lake City, UT, USA), which simultaneously detects 30 species, 3 organism groups, and 10 antimicrobial resistance genes associated with bloodstream infections.^27^ Per this protocol, a critical blood culture value prompted the laboratory to call the patient’s care team, usually the nurse,^12^ to alert them about the positive culture and that BCID results for the patient would become available within the upcoming 6 hours. No second call was made to the nurse. The pharmacist would receive the BCID results through inbox messages and discuss the necessary adjustments to antibiotic utilization with the physician based on these results. Monitoring of antibiotic utilization under this protocol was from March 1, 2022, to October 31, 2023 (20 months; **time 1**).

From November 1, 2023, to August 31, 2025 (22 months; **ASI 2, time 2**), most microbiology samples collected at Touro were sent to the University Medical Center (UMC), another institution within the LCMC Health system in New Orleans, for laboratory testing. Blood culture remained at Touro until positive. When the blood culture turned positive, the bottle was removed from the automated instrument and had both Gram stain and BCID2 performed per protocol.^27^ The critical value was reported to the patient’s care team, and then the positive blood culture bottle was sent to UMC for further workup, including plating, identification, and susceptibility testing. The other cultures, such as urine cultures, respiratory tract cultures, or wound cultures, were sent to UMC, causing concerns over possible delays in processing times of these cultures compared to turnaround times when all microbiology laboratory testing was performed at Touro.

### Data collection and study outcomes

We evaluated days of antibiotic therapy for antipseudomonal beta-lactams, anti-MRSA antibiotics, fluoroquinolones, and ceftriaxone; days of total antibiotic therapy; and days of intravenous antibiotic administration at the hospital. These were reported at baseline (19 months), during 10 months of COVID-19-dictated antibiotic utilization, and during 14 months of MRSA/PCR testing at Touro (ASI 1), 20 months of BCID at Touro (ASI 2, time 1), followed by 22 months of BCID and other cultures performed at UMC (ASI 2, time 2).

### Statistical analysis

Days of antibiotic therapy were calculated per 1,000 patient-days attended, captured in EPIC, and exported into an Excel spreadsheet. Data analysis was performed using SPSS statistical software (IBM SPSS Statistics 25). Days of antibiotic therapy and of intravenous antibiotic administration were compared using the Kruskal-Wallis test when comparing more than two observation periods or the Mann-Whitney *U* test when comparing two observation periods, with statistical significance set at *P* < .05.

## Results

**Figure 1.**
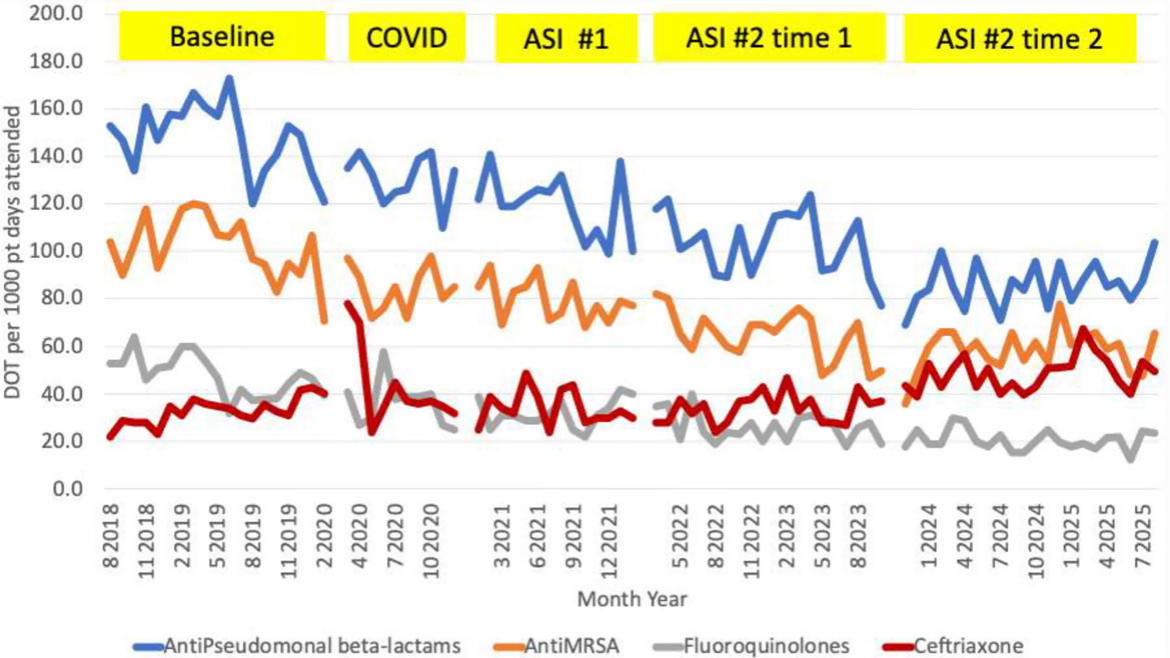
Monthly days of antibiotic therapy per 1,000 patient-days, Touro Infirmary, New Orleans, Louisiana.

Figure 1 shows the monthly distributions of days of antibiotic therapy per 1,000 patient-days present at the hospital for antipseudomonal beta-lactams, anti-MRSA antibiotics, fluoroquinolones, and ceftriaxone during the 85-month observation period.

Significant changes in antimicrobial utilization were observed during the study period (Table 1). Days of therapy for antipseudomonal beta-lactams, anti-MRSA, and fluoroquinolones consistently and significantly decreased from baseline to ASI 2, time 2.

**Table 1.** Days of antibiotic therapy per 1,000 patient-days, Touro Infirmary, New Orleans, Louisiana. Data are medians [IQR]

		Anti pseudomonal beta-lactams	Anti MRSA	Fluoro-quinolones	Vancomycin	Meropenem	Ceftriaxone	Total antibiotic	Intravenous antibiotic
**Baseline**		149.0 [134.0–158.0]	104.0 [93.0–112.3]	47.0 [39.5–53.0]	87.0 [80.1–93.0]	17.7 [14.4–20.0]	32.8 [29.0–36.0]	600.3 [580.5–629.4]	436.2 [421.0–465.3]
**COVID-19 protocol**		133.5 [123.7–139.7]	85.0 [75.0–91.0]	38.5 [27.0–40.2]	71.5 [68.5–80.5]	21.0 [14.7–27.0]	36.5 [33.5–51.2]	550.0 [512.2–590.7]	383.0 [375.7–413.7]
**ASI 1**		120.5 [107.2–127.5]	78.0 [70.7–85.5]	31.0 [28.0–37.5]	71.5 [61.0–76.2]	18.5 [13.7–21.7]	32.5 [29.5–39.7]	527.0 [512.7–546.0]	384.5 [361.5–403.5]
**ASI 2**	**Time 1**	104.0 [90.5–115.0]	66.0 [58.2–72.0]	26.5 [20.2–30.0]	57.0 [52.2–64.7]	16.0 [12.2–19.0]	34.5 [28.0–38.0]	477.2 [457.7–504.7]	351.1 [328.7–370.2]
	**Time 2**	85.0 [79.5–95.5]	60.4 [53.2–65.6]	19.9 [18.0–23.8]	54.2 [47.7–57.6]	10.5 [7.0–14.2]	50.3 [43.0–53.2]	492.0 [470.8–505.1]	359.0 [345.2–376.2]
*P* value^*^		**.003**	**.001**	**.006**	**.001**	.179	.069	**.024**	**.002**
*P* value^**^		**<.0001**	**<.0001**	**<.0001**	**<.0001**	**<.0001**	**<.0001**	**<.0001**	**.003**
***P* value** ^**¶**^		**<.0001**	**.039**	**.003**	.118	**<.0001**	**<.0001**	.571	.217
***P* value** ^**§**^		**<.0001**	**<.0001**	**<.0001**	**<.0001**	**<.0001**	**<.0001**	**<.0001**	**<.0001**

* Mann-Whitney *U* test for comparisons between Baseline and the COVID-19 period.

** Kruskal-Wallis test for comparisons between ASI 1 and ASI 2 (times 1 and 2).

^¶^Mann-Whitney *U* test for comparisons between times 1 and 2 of ASI 2.

^§^Kruskal-Wallis test for comparisons between the 5 observation periods.

ASI, Antimicrobial Stewardship Intervention; IQR, interquartile range; MRSA, methicillin-resistant *Staphylococcus Aureus*.

*P* value, boldface denotes a statistically significant difference.

Days of therapy for vancomycin decreased significantly from baseline to the COVID-19 period (medians: 87.0 days vs 71.5 days; *P* = .001), then decreased significantly (*P* < .0001) from ASI 1 (median: 71.5 days) to ASI 2, time 2 (median: 54.2 days). However, the decrease between ASI 2, time 1 (median: 57.0 days) and time 2 (median: 54.2 days) was not statistically significant (*P* = .118).

Days of therapy for meropenem did not change significantly from baseline to the COVID-19 period (medians: 17.7 days vs 21.0 days; *P* = .179) but decreased significantly through ASI 2, time 2 (median: 10.5 days; *P* < .0001).

Days of therapy for ceftriaxone increased significantly from ASI 1 (median: 32.5 days) to ASI 2, time 2 (median: 50.3 days) (*P* < .0001).

Total antibiotic therapy consistently decreased from a median of 600.3 days at baseline to a median of 477.2 days during ASI 2, time 1 (*P* < .0001), but these did not change significantly to time 2 (median: 492.0 days; *P* = .571).

Days of intravenous antibiotic administration significantly decreased (*P* = .002) from baseline (median: 436.2 days) to the COVID-19 period (median: 383.0 days) and decreased again significantly (*P* = .003) from ASI 1 (median: 384.5 days) to ASI 2, time 1 (median: 351.1 days). The slight increase from ASI 2, time 1 to time 2 was not significantly different (medians: 351.1 days to 359.0 days, respectively; *P* = .217).

## Discussion

### Baseline and COVID-19 monitoring

We found a statistically non-significant increase in days of meropenem and ceftriaxone utilization at Touro during the COVID-19 pandemic, which was consistent with increased utilization of meropenem and ceftriaxone, along with other broader-spectrum antibiotics, observed during the pandemic worldwide.^28,29^ For example, at one tertiary care University hospital in Turkey, the numbers of defined daily doses per 100 days of bed increased 82.8% for meropenem and 23.1% for ceftriaxone between the prepandemic 12-month period going from March 11, 2019, to March 10, 2020, and the pandemic 12-month period going from March 11, 2020, to March 10, 2021, respectively.^29^ Days of utilization of all other antibiotics and days of intravenous antibiotic administration at Touro significantly decreased from baseline to the COVID-19 period. These improved patient outcomes can be attributed to the AS activities implemented at the hospital since the ASP team was solidly established there.

### ASI 1

Per the MRSA PCR package insert, a positive test result does not necessarily indicate the presence of viable organisms; it is, however, presumptive of MRSA or *S. aureus*. Also, a negative test result does not preclude MRSA or *S. aureus* nasal colonization. At Touro, vancomycin remains the antibiotic of choice to treat MRSA infections.^30^ In this study, we reported its utilization separately from the other anti-MRSA drugs simply because it was the most frequently used in this class, but its days of utilization are also included in the computation of days of utilization of all anti-MRSA drugs we reported. Other anti-MRSA less frequently used at Touro, because they are restricted, were daptomycin, linezolid, and ceftaroline. After the implementation of the MRSA PCR testing, our data showed a significant decrease in vancomycin utilization and a significant increase in ceftriaxone utilization at the hospital, which indicates that this ASI changed antibiotic use in an appropriate and expected fashion.

### ASI 2, time 1

The introduction of BCID at the local laboratory decreased the time required for blood culture and genotyping resistance data to become available. Our data showed that with this intervention coupled with all other AS activities at Touro, vancomycin utilization continued to decline, and meropenem utilization also decreased. These results suggest a more rapid de-escalation of broad-spectrum antibiotics at the hospital.^16–21^

### ASI 2, time 2

When samples collected at Touro for urine cultures, respiratory tract cultures, wound cultures, and positive blood culture bottles were sent to UMC for testing and further workup, this caused concerns over possible delays in results. Our data indicated that days of total antibiotic therapy increased slightly, but not significantly. We did not find any published study that evaluated the impact of moving the clinical microbiology laboratory on antibiotic utilization outcomes.

During the study period, we found that antibiotic utilization for antipseudomonal beta-lactams, anti-MRSA antibiotics, and fluoroquinolones decreased significantly at Touro. Because ceftriaxone is the de-escalation antibiotic of choice at the hospital, days of therapy for this antibiotic significantly increased, as we expected. Although ceftriaxone utilization increased over time, this increase did not offset the decreases achieved with all other antibiotics, and as a result, total antibiotics utilization at the hospital showed a significant decrease from baseline to ASI 2, time 1.

The antipseudomonal beta-lactams most frequently used are cefepime, ceftazidime, imipenem, meropenem, and piperacillin/tazobactam, although there are others in this class of antibiotics.^31,32^ At Touro, with weekday reviews for their appropriateness, the most frequently used was piperacillin/tazobactam, followed by cefepime, then meropenem. However, we reported meropenem utilization separately from the more frequently used antipseudomonal beta-lactams because it was restricted, even during the COVID-19 period when its utilization slightly increased.

Tracking fluoroquinolone use is aimed at reducing their utilization over time out of concerns about known adverse events as well as development of antimicrobial resistance to this potent, broader-spectrum class of antibiotics, which includes ciprofloxacin, levofloxacin, and moxifloxacin, among others.^5–7,33^ The fluoroquinolones that were used at Touro were ciprofloxacin for urinary tract infection and levofloxacin for pneumonia. Through education and feedback, the ASP pushed for the use of any alternatives that could be used in urinary tract infection instead of ciprofloxacin and azithromycin and ceftriaxone in pneumonia instead of levofloxacin. We found that fluoroquinolone utilization at Touro decreased significantly and consistently during the observation period, which may represent changing prescription practices that may be associated with AS-targeted education and feedback regarding this class of antibiotics.

In addition to the duration of antimicrobial therapy, AS activity includes selecting the appropriate route of antibiotic administration in hospitalized patients.^24^ We evaluated days of intravenous antibiotic administration in relation to AS activities/interventions implemented and monitored at the hospital. Intravenous antibiotic administration decreased steadily from baseline through ASI 2, time 1.

There are several limitations to this study. Days of antibiotic utilization we reported were aggregated for the whole hospital; thus, antibiotic utilization at different units/wards of the hospital are confounded therein. The clinical indications for which these antibiotics were prescribed were not linked to the data we report. We therefore cannot determine the conditions for which these antibiotics were prescribed empirically to patients. As recorded, our data cannot be used to determine whether or how other patients’ outcomes such as hospital or infection-related length of stay, improvement of clinical syndromes, or in-hospital mortality, may have been or not affected by AS activities/interventions. Despite these limitations and maybe some others that are inherent to the outcome measures we reported, our study clearly showed that clinical microbiology laboratories integrated into ASP are associated with reductions in days of antibiotic utilization and of intravenous antibiotic administration at Touro Infirmary in New Orleans.^23^

In conclusion, our data indicated that AS activities complemented by MRSA screening (ASI 1) and BCID (ASI 2) decreased utilization of vancomycin and of broad-spectrum antibiotics at Touro Infirmary. We need to continue to monitor the long-term trend of antibiotic utilization to determine whether days of total antibiotic utilization and days of intravenous antibiotic administration at the hospital have reached a plateau. Future studies should also examine antibiotic utilization at different units/wards of the hospital, the indications for which patients are prescribed antibiotics empirically, hospital- or infection-related length of stay,^18–20,22^ in-hospital mortality,^20,21,23^ and hospital or infection-related costs.^18,19,21,22^
